# Effects of Chronic Central Arginine Vasopressin (AVP) on Maternal Behavior in Chronically Stressed Rat Dams

**DOI:** 10.3390/brainsci2040589

**Published:** 2012-11-07

**Authors:** Alexander J. Coverdill, Megan McCarthy, Robert S. Bridges, Benjamin C. Nephew

**Affiliations:** Department of Biomedical Sciences, Cummings School of Veterinary Medicine, Tufts University, 200 Westboro Rd, North Grafton, MA 01536, USA; Email: megan.mccarthy@aya.yale.edu (M.M.); robert.bridges@tufts.edu (R.S.B.); bcnephew@aol.com (B.C.N.)

**Keywords:** maternal behavior, maternal care, maternal aggression, AVP, vasopressin, social stress, lactation

## Abstract

Exposure of mothers to chronic stressors during pregnancy or the postpartum period often leads to the development of depression, anxiety, or other related mood disorders. The adverse effects of mood disorders are often mediated through maternal behavior and recent work has identified arginine vasopressin (AVP) as a key neuropeptide hormone in the expression of maternal behavior in both rats and humans. Using an established rodent model that elicits behavioral and physiological responses similar to human mood disorders, this study tested the effectiveness of chronic AVP infusion as a novel treatment for the adverse effects of exposure to chronic social stress during lactation in rats. During early (day 3) and mid (day 10) lactation, AVP treatment significantly decreased the latency to initiate nursing and time spent retrieving pups, and increased pup grooming and total maternal care (sum of pup grooming and nursing). AVP treatment was also effective in decreasing maternal aggression and the average duration of aggressive bouts on day 3 of lactation. Central AVP may be an effective target for the development of treatments for enhancing maternal behavior in individuals exposed to chronic social stress.

## 1. Introduction

Depression, chronic anxiety, and other mood disorders can have negative consequences on mothers as well as their offspring, and the occurrence of these disorders during lactation has been associated with impaired child growth and development [1,2]. While the adverse effects of mood disorders are often mediated through maternal behavior [3], few studies investigating depression focus on maternal females and offspring care. Exposure to various stressors during pregnancy or the postpartum period are often linked to the development of mood disorders [4,5,6,7,8,9,10,11,12,13], and in humans, chronic exposure to psychosocial stressors, such as social conflict, is one of the strongest predictors of postpartum depression [14]. 

While numerous animal models of stress-induced depression and anxiety disrupt maternal behavior, most involve significant physiological components and may not replicate the effects of chronic psychosocial stressors in humans. In rodents for example, chronic restraint stress or exposure to wet bedding decreases maternal aggression [15] and increases maternal care [16]. In addition, most animal models fail to replicate high levels of social conflict or low levels of social support that are often implicated in postpartum depression in humans [5,7]. Given that acute exposure to a novel male intruder elicits robust behavioral and endocrine stress responses in lactating rats [17,18,19,20], Nephew and Bridges [21] established a chronic social stress (CSS) paradigm where lactating females were repeatedly exposed to novel male intruders. CSS exposure results in decreased maternal care and saccharin preference, decreased milk intake by pups, decreased growth of both dams and pups, and increased aggression towards the novel intruder male [21,22]. Further study of the adult offspring of stressed dams indicates that CSS is a potent early life stressor that decreases nursing efficiency. Decreased nursing efficiency is a behavioral effect associated with increased basal plasma corticosterone and decreased basal plasma estradiol, as well as decreased hypothalamic vasopressin (AVP), oxytocin (OXT) and prolactin (PRL) activity [22,23]. Having incorporated a social stressor and eliciting behavioral and physiological responses similar to those seen in human mood disorders, the CSS paradigm serves as a suitable model to test the effectiveness of novel treatments for stress induced disorders that affect maternal behavior. 

The various roles of neuropeptide hormones in the regulation and expression of maternal behavior in both rats and humans have been studied for decades, and several have been linked to the etiology of stress related mood disorders [24,25]. Arginine vasopressin (AVP) is a neuropeptide hormone implicated in the regulation of several social behaviors, including both maternal care and aggression [26,27]. While maternal care plays an intuitive role in promoting offspring survival, maternal aggression is also critical for the protection of altricial young [28,29,30,31,32,33,34]. AVP modulates aggressive displays in numerous rodent species [35,36,37,38,39,40,41,42,43,44] and the intensity of maternal aggressive behavior has been shown to vary due to hormonal fluctuations over the course of lactation and across litters [19,20,45,46,47]. While AVP promotes offensive aggression during social encounters in male rodents [36,37], AVP has been shown to have both stimulatory [48] and inhibitory [19,49] effects on the display of maternal aggression in lactating female rats, depending on the target brain region. AVP has also been shown to decrease conspecific aggression in nulliparous female hamsters [44]. AVP possibly mediates maternal behavior by acting through V1a receptors to modulate sensory processing [50], as treatment with AVP increases maternal care [19,51], and AVP V1a receptor antagonists impair maternal memory [52] and reduce nursing and retrieval of pups [19,53].

The objective of this investigation was to test the effectiveness of AVP as a novel treatment for stress induced disorders that affect maternal behavior. It was postulated that chronic central AVP infusion would alter maternal behavior and growth patterns in dams and their pups exposed to the CSS rodent model for postpartum depression and anxiety. More specifically, it was hypothesized that AVP treatment would decrease aggression towards a novel male intruder, increase maternal care towards offspring, and prevent attenuated growth in both the dam and pups.

## 2. Results and Discussion

### 2.1. Maternal Care Testing

**Figure 1:**
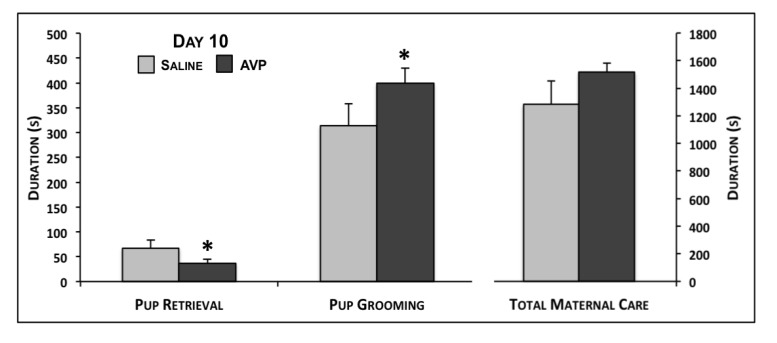
Mean + SEM duration values for pup retrieval, pup grooming and total maternal care (sum of pup grooming and nursing) on day 10 of lactation during a 30 min maternal care observation test of chronic social stressed dams treated with either saline or AVP. * indicates a significant difference between treatments (*p* ≤ 0.05).

There were no overall effects of AVP treatment on any behavioral variable, but there were several behaviors significantly affected by lactation day (pup grooming, nursing, nursing latency, total maternal care, nesting and self grooming; all *p*-values < 0.02, repeated measures ANOVA). However, the primary focus of the current study is to investigate the effects of AVP on maternal behavior; therefore, the effects of lactation day (time) will not be discussed in detail. During maternal care testing on day 10, the time required to retrieve all 8 pups was decreased in AVP dams (36.8 ± 8.2 *vs.* 67.1 ± 16.6 s, *t*_37_ = 1.85, *p* = 0.04; Figure 1), and the duration of pup grooming was higher in AVP animals compared to saline (399.3 ± 29.8 *vs.* 313.8 ± 44.7 s, *t*_37_ = −1.65, *p* = 0.05; Figure 1). In addition, there was a trend towards increased total maternal care (sum of pup grooming and nursing) in AVP dams (1516.3 ± 65.1 *vs.* 1283.1 ± 169.1 s, *t*_37_ = −1.53, *p* = 0.07). There were no differences between the saline and AVP groups in any measure of behavior during the maternal care tests on lactation days 3 or 17 (Table 1).

**Table 1:** Means ± SEM behavioral data during 30-min maternal care tests on lactation days 3, 10 and 17. Retrieval data were not collected on day 17 as pups were often active and moving about the cage. Data in bold represent significant differences between saline and AVP treated animals.

Behavior Variable ^a^	Day 3			Day 10			Day 17		
	Saline (*n* = 16)	AVP (*n* = 26)	*p*	Saline (*n* = 14)	AVP (*n* = 25)	*p*	Saline (*n* = 13)	AVP (*n* = 25)	*p*
Pup Grooming	139.9 ± 33.5	162.7 ± 17.4	0.23	**313.8 ± 44.7**	**399.3 ± 29.8**	**0.05**	362.6 ± 43.7	328.5 ± 29.3	0.26
Nursing	744.2 ± 118.5	781.8 ± 74.4	0.39	969.2 ± 135.2	1116.9 ± 62.2	0.13	884.5 ± 140.1	926.1 ± 80.0	0.39
Nursing Latency	684.7 ± 123.7	649.8 ± 73.7	0.40	544.3 ± 121.1	411.0 ± 44.1	0.11	640.4 ± 135.5	580.0 ± 55.4	0.31
Total Maternal Care	884.1 ± 137.5	944.5 ± 81.1	0.34	1283.1 ± 169.1	1516.3 ± 65.1	0.07	1247.0 ± 171.5	1254.6 ± 90.9	0.48
Nesting	124.7 ± 17.1	144.0 ± 19.4	0.25	75.4 ± 14.0	69.2 ± 8.5	0.34	30.7 ± 12.3	41.6 ± 8.0	0.22
Self Grooming	160.0 ± 34.5	169.6 ± 23.9	0.41	65.6 ± 13.0	81.2 ± 17.4	0.27	79.9 ± 19.1	65.9 ± 13.6	0.28
Retrieval	55.8 ± 11.1	51.0 ± 7.0	0.35	**67.1 ± 16.6**	**36.8 ± 8.2**	**0.04**	--	--	--
Full Retrieval	415.7 ± 121.5	357.7 ± 49.6	0.31	397.1 ± 111.9	352.8 ± 76.5	0.37	--	--	--
Activity	100.1 ± 13.6	102.4 ± 12.7	0.45	88.1 ± 12.5	82.3 ± 7.4	0.34	108.6 ± 14.1	103.3 ± 8.0	0.36

^a^ Behavioral durations are presented in seconds (s).

### 2.2. Maternal Aggression Testing

**Figure 2:**
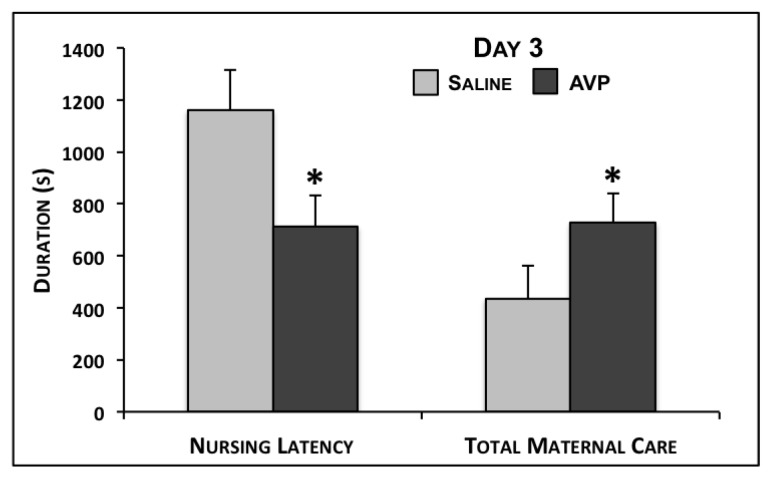
Mean + SEM duration values for nursing latency and total maternal care (sum of pup grooming and nursing) on day 3 of lactation during a 30 min maternal aggression observation test of chronic social stressed dams treated with either saline or AVP. * indicates a significant difference between treatments (*p* ≤ 0.05).

**Figure 3:**
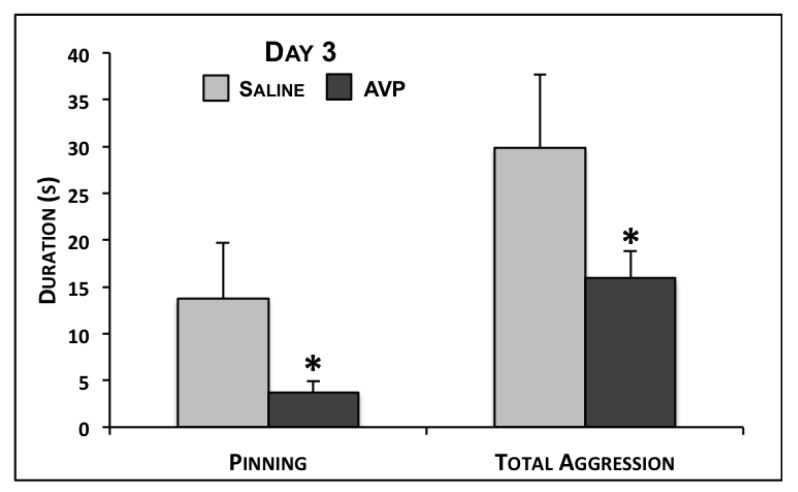
Mean + SEM duration values for pinning and total aggression (sum of attacks, pinning, kicking and biting) on day 3 of lactation during a 30 min maternal aggression observation test of chronic social stressed dams treated with either saline or AVP. * indicates a significant difference between treatments (*p*≤ 0.05).

Several behaviors were significantly affected by lactation day (pup grooming, total maternal care, self-grooming and locomotor activity; all *p*-values <0.02, repeated measures ANOVA). However, the primary focus of the current study is to investigate the effects of AVP on maternal behavior; therefore, the effects of lactation day (time) will not be discussed in detail. During the maternal aggression tests on lactation day 3, latency to initiate nursing was decreased in AVP dams (714.2 ± 117.2 *vs.* 1160.9 ± 153.5 s, *t*_40_ = 2.33, *p* = 0.01; Figure 2) and total maternal care (sum of pup grooming and nursing) was increased in AVP treated animals (727.8 ± 113.2 *vs.* 435.1 ± 128.0 s, *t*_40_ = −1.66, *p* = 0.05; Figure 2). The duration of pinning was lower in the AVP treated group (3.7 ± 1.1 *vs.* 13.7 ± 6.0 s, *t*_40_ = 2.04, *p* = 0.02; Figure 3), resulting in a lower duration of total aggression (15.9 ± 2.9 *vs.* 29.9 ± 7.8 s, *t*_40_ = 1.97, *p* = 0.03; Figure 3) and shorter average aggressive bout duration than saline dams (0.5 ± 0.1 *vs.* 0.8 ± 0.1 s, *t*_40_ = 2.26, *p* = 0.01). Locomotor activity duration was decreased in the AVP group on day 3 (63.4 ± 6.0 *vs.* 95.9 ± 11.6 s, *t*_40_ = 2.75, *p* < 0.01). There were no differences in behavior between the saline and AVP groups during maternal aggression tests on lactation days 10 or 17 (Table 2).

**Table 2:** Means ± SEM behavioral data during 30-min maternal aggression tests on lactation days 3, 10 and 17. Data in bold represent significant differences between saline and AVP treated animals.

Behavior Variable ^a^	Day 3			Day 10			Day 17		
	Saline (*n* = 16)	AVP (*n* = 26)	*p*	Saline (*n* = 14)	AVP (*n* = 25)	*p*	Saline (*n* = 13)	AVP (*n* = 25)	*p*
Pup Grooming	14.8 ± 5.2	27.1 ± 6.1	0.08	188.7 ± 43.4	169.1 ± 26.1	0.34	138.0 ± 45.2	117.7 ± 24.5	0.33
Nursing	420.3 ± 127.4	700.6 ± 110.5	0.06	880.7 ± 176.3	741.7 ± 108.6	0.24	572.4 ± 110.4	559.5 ± 90.0	0.46
Nursing Latency	**1160.9 ± 153.5**	**714.2 ± 117.2**	**0.01**	680.4 ± 158.5	681.2 ± 126.8	0.50	887.3 ± 115.7	814.6 ± 135.3	0.36
Total Maternal Care	**435.1 ± 128.0**	**727.8 ± 113.2**	**0.05**	1069.4 ± 206.7	910.9 ± 126.7	0.25	710.3 ± 143.0	677.2 ± 106.1	0.43
Nesting	23.7 ± 9.3	67.3 ± 22.8	0.08	65.3 ± 29.5	46.4 ± 12.7	0.25	28.5 ± 12.9	30.5 ± 8.1	0.44
Self Grooming	165.5 ± 33.1	165.0 ± 18.7	0.50	135.5 ± 22.2	146.5 ± 24.2	0.38	109.7 ± 20.6	105.3 ± 9.8	0.41
Activity	**95.9 ± 11.6**	**63.4 ± 6.0**	**<0.01**	69.2 ± 12.6	76.8 ± 8.3	0.30	98.3 ± 12.4	92.8 ± 11.2	0.38
Attacking	12.3 ± 2.2	8.9 ± 1.6	0.10	7.6 ± 1.7	10.8 ± 1.3	0.08	6.4 ± 1.0	7.4 ± 1.2	0.29
Biting	1.5 ± 0.5	1.9 ± 0.8	0.36	0.9 ± 0.5	1.3 ± 0.6	0.30	1.4 ± 0.5	1.2 ± 0.6	0.40
Kicking	2.3 ± 0.5	1.4 ± 0.4	0.08	1.2 ± 0.4	2.2 ± 0.8	0.19	0.3 ± 0.1	1.1 ± 0.4	0.06
Pinning	**13.7 ± 6.0**	**3.7 ± 1.1**	**0.02**	1.3 ± 0.6	15.9 ± 10.6	0.16	1.0 ± 0.7	9.5 ± 6.0	0.16
Total Aggression	**29.9 ± 7.8**	**15.9 ± 2.9**	**0.03**	11.0 ± 2.2	30.3 ± 12.0	0.12	9.2 ± 1.4	19.2 ± 6.9	0.15
Aggression Bout	**0.8 ± 0.1**	**0.5 ± 0.1**	**0.01**	0.6 ± 0.1	0.8 ± 0.1	0.23	0.5 ± 0.0	0.8 ± 0.2	0.17
Attack Latency	62.5 ± 21.5	44.8 ± 12.3	0.22	54.6 ± 17.0	76.0 ± 29.8	0.31	209.6 ± 99.6	226.3 ± 78.0	0.44

^a^ Behavioral durations are presented in seconds (s).

### 2.3. Growth

There were no differences in mean body weight between saline and AVP treated dams on lactation day 3, (326.1 ± 6.2 *vs.* 319.4 ± 6.4 g, *p* = 0.25), day 10 (348.6 ± 6.5 *vs.* 342.2 ± 6.1 g, *p* = 0.26) or day 17 (364.5 ± 6.1 *vs.* 353.9 ± 4.9 g, *p* = 0.10). Similarly there were no differences in mean body weight between the pups of saline and AVP treated dams on day 3 (7.8 ± 0.3 *vs.* 8.5 ± 0.3 g, *p* = 0.08), day 10 (23.9 ± 0.5 *vs.* 25.1 ± 0.5, *p* = 0.08) or day 17 (44.8 ± 0.8 *vs.* 45.2 ± 0.8 g, *p* = 0.35). Absolute weight gain (in grams) and percent weight gain (relative to day 3) were similar for both dams and pups of saline and AVP groups on day 10, but not day 17 (Table 3). While the absolute weight gained from day 3 to 17 was similar for pups of saline and AVP dams (37.0 ± 0.7 *vs.* 36.8 ± 0.6 g, *p* = 0.42), the percent weight increase was larger for saline than AVP pups (483.0% ± 18.9% *vs.* 443.2% ± 11.8%, *t*_36_ = 1.86, *p* = 0.04; Table 3). Growth measures recorded in the current study are consistent with data collected from chronic social stressed dams in previous work [21], where CSS attenuated the growth of both dams and pups compared to non-stressed control animals.

**Table 3:** Means ± SEM of absolute (g) and percent (%) body weight gain relative to lactation day 3 for Saline and AVP dams and pups on days 10 and 17. Data in bold represent significant differences between saline and AVP treated animals.

Age	Day 10			Day 17		
	Saline (*n* = 14)	AVP (*n* = 25)	*p*	Saline (*n* = 13)	AVP (*n* = 25)	*p*
Dams (g)	22.5±3.6	22.8±2.8	0.47	38.5±5.1	34.5±3.4	0.26
Dams (%)	7.0 ± 1.1	7.3 ± 1.0	0.42	12.0 ± 1.6	11.2 ± 1.2	0.36
Pups ^a^ (g)	16.2 ± 0.4	16.6 ± 0.3	0.17	37.0 ± 0.7	36.8 ± 0.6	0.42
Pups ^a^ (%)	211.4 ± 9.3	200.0 ± 5.2	0.13	**483.0 ± 18.9**	**443.2 ± 11.8**	**0.04**

^a^ Pup body weight changes represent the average measure of 8 pups in each litter.

### 2.4. Discussion

The objective of this study was to test the effectiveness of AVP as a novel treatment for enhancing maternal behavior and growth in dams and litters exposed to chronic social stress (CSS). The exposure of saline control dams to a novel male intruder resulted in behavioral and growth responses consistent with stressed dams in the other studies [21,22], thus the surgical procedures utilized for central AVP administration did not appear to have adverse implications. The CSS protocol is more ethologically and clinically relevant as a model for human depression and anxiety disorders than other animal models of stress-induced depression which involve robust physiological confounds and/or have inconsistent effects. The current results support the hypotheses that chronic central AVP promotes maternal care and decreases maternal aggression in dams exposed to CSS, but do not support the hypothesis that AVP increases pup growth.

To date, AVP research has focused primarily on male-typical social behaviors, including aggression and pair-bond formation, with less attention devoted to the effects of neuropeptides on maternal social behavior [54]. The observation that the results of this study conflict with the established male-biased literature on the role of AVP in chronic stress associated disorders such as depression and anxiety is significant [25,55,56,57,58,59,60,61,62]. Considering that AVP differentially affects social communication in men and women [63], our results indicate that data from males concerning this topic may not be applicable to females. Vasopressin based treatments aimed at attenuating depression and anxiety in males may have adverse effects on maternal care and/or aggression.

AVP treatment significantly affected maternal care behaviors during maternal care testing (dams alone with pups) and when dams were faced with an acute stressor during maternal aggression testing (novel male intruder). During maternal care testing on day 10 of lactation, AVP dams significantly decreased the amount of time spent retrieving pups back to the nest (more efficient retrieval) and increased pup grooming. Similarly, during the maternal aggression testing on lactation day 3, AVP treated dams initiated nursing more quickly (decreased nursing latency) than saline dams, and AVP dams exhibited a significant increase in total maternal care behavior. These results are consistent with previous work investigating the neuroendocrine and behavioral parameters of rats selectively bred for high anxiety-related behavior (HAB). While it has been shown that the responsiveness of the hypothalamo-pituitary-adrenal (HPA) axis is attenuated during the natural progression of pregnancy and lactation [64,65], HAB dams exhibit a hyper-responsiveness of the HPA-axis [66] due to higher endogenous secretion of AVP [67,68,69,70]. Similar to the AVP dams in the present study, HAB dams exhibit more direct pup contact and spend less time retrieving pups back to the nest than dams with lower endogenous AVP bred for low-anxiety related behavior (LAB) [71]. In addition, when given icv infusions of AVP, LAB dams increase maternal behavior [51] and administration of a central V1a antagonist decreases maternal care during aggression tests [19] and impairs maternal memory [52]. We propose that the faster initiation of nursing and the increase in maternal care behavior on day 3 led to the decrease in general locomotor activity observed in AVP dams, and that AVP did not directly affect locomotor activity. Collectively, the data support the hypothesis that chronic icv AVP increases maternal care by stimulating interactions between CSS exposed dams and their pups. 

The significant effects of chronic icv AVP on maternal aggression behavior support the hypothesis that AVP administration decreases maternal aggression towards a novel male intruder. While maternal aggression is typically highest during early lactation [20,45,46], infusion of AVP on lactation day 3 significantly reduced total maternal aggression (sum of attacking, biting, kicking and pinning), with a marked reduction of pinning behavior as compared to saline treated dams. Similarly, the average length of an aggressive bout was reduced in AVP treated dams compared to saline animals. It has been shown that dams exposed to chronic social stress increase the average duration of aggressive bout encounters with a male intruder [21], and the combination of the overall decrease in maternal aggression and shorter bout duration in AVP dams support an inhibitory role for this neuropeptide on maternal aggression. These effects of acute AVP treatment (only present on day 3) are consistent with other work reporting decreased aggression in non-stressed maternal rats and virgin female hamsters given central AVP injections [44,49]. When combined with the fact that AVP mRNA is decreased in the supraoptic and paraventricular nuclei during early lactation [72] coincident with high levels of maternal aggression [45], the results of this study underscore AVP’s role in regulating maternal aggression. 

The absence of AVP effects on any behavioral measure during maternal care testing on lactation day 3 may suggest that AVP has no acute effect on maternal care. However, the positive effects of AVP on both maternal care and aggression during the maternal aggression tests on day 3 suggest otherwise. The consistent effects of AVP on maternal care both during typical dam-pup interactions as well as during the exposure to a social stressor indicate that the treatment was effective after 24 h (day 3) and one week (day 10). It is likely that the lack of effect on maternal care during aggression testing on day 10 was due to the fact that the maternal care test immediately preceded the maternal aggression test. The lack of significant treatment effects on lactation day 17 may be due to a down-regulation of central endogenous AVP production in response to the continuous infusion, resulting in a dampened effect of the exogenous treatment. Conversely, the lack of significant effects during late lactation may be due to a ceiling effect [73], where AVP may have no impact during a period when maternal care is already typically low [19,20]. Other possible explanations are that the V1a receptors were down-regulated in response to the AVP infusion, or that central oxytocin (OXT) activity was similarly affected. The expression of both AVP and OXT neuropeptides have been associated with decreases in maternal aggression related to parity and/or stage of lactation [20]. The results of this study suggest that dams may become desensitized to AVP infusions during mid-late lactation; thus, AVP treatment had no effect on maternal aggression on day 10, or on maternal care and/or aggression on day 17. 

The hypothesis that AVP treatment would increase growth in dams and/or pups following CSS exposure was not supported by the growth profiles. There was no difference in body weight or weight gain between control and AVP animals on either day 3 or 10 of lactation. Interestingly, while there was no difference in absolute weight gain between pups of saline and AVP treated dams on day 17, the percent weight increase from day 3 to day 17 was less in AVP litters compared with controls. Similar effects are seen in humans, where infants of mothers suffering from postnatal depression suffered poorer growth than infants of non-depressed mothers [74], presumably due to the negative effects of stress on lactation efficiency and milk quality [75]. Chronic exposure to psychosocial stressors result in elevated expression of AVP, that, when combined with a hyperactivation of the HPA-axis, leads to decreased growth [76]. It is possible that the increased maternal care during early and mid lactation effectively compensated for potential adverse effects of AVP infusion on growth. Similar to the effects of AVP on maternal behavior, the decreased growth of AVP pups on day 17 may be attributed to an overall desensitization of dams to AVP infusions. However, given the similarities between absolute pup weight gain and the average pup weight between groups on day 17, the decreased percent weight gain does not appear to be physiologically substantial. When also considering the growth data from days 3 and 10, it is concluded that AVP treatment did not significantly affect dam or pup growth. 

## 3. Experimental Section

### 3.1. Animals

Female Sprague-Dawley rats (175–200 g) were obtained from Charles River Laboratories (Wilmington, MA) and maintained in temperature (21–25 °C) and light (14:10 light-dark cycle, lights on at 5:00 a.m.) controlled rooms. Food (Purina rat chow) and water were provided *ad libitum* throughout the study. Rats were mated by placing 2 females with 1 male for 8 days. Following mating, females were housed communally (3 per cage) until one day prior to parturition when each female was housed individually. Normal maternal behavior (retrieval, grouping and nursing of pups following parturition) was observed in all animals on lactation day 1, litters were culled to 8 pups (4 male/4 female), and dams were randomly assigned to saline control, low dose AVP, or high dose AVP groups. On lactation day 2, rats were anesthetized with isoflurane and implanted with chronic guide cannulae directed into the right lateral ventricle (placements were confirmed with India ink). ALZET (Cupertino, CA) osmotic pumps (model 2002; reservoir volume of 200 μL, flow rate of 0.5 μL/h) were primed and connected to the chronic guide cannulae and implanted subcutaneously in the upper back region. The pumps contained one of two doses of AVP (0.5 or 5.0 ng/h, Sigma), or saline vehicle. While the initial group sizes for control, low dose AVP and high dose AVP were 16, 12 and 14, respectively, intruder males attacked and killed the pups of 4 dams during the study. As a result, these dams were removed from subsequent maternal behavior testing (decreasing group sizes to 14, 12 and 13 for testing on lactation day 10 and 13, 12 and 13 on day 17). Animals in this study were maintained in accordance with the guidelines of the Committee of the Care and Use of Laboratory Animals Resources, National Research Council, and the research protocol was approved by the Tufts University Institutional Animal Care and Use Committee. 

### 3.2. Chronic Social Stress Paradigm

The CSS protocol used in the current study was closely based on the 2011 study by Nephew and Bridges [21]. On lactation days 3–17, an intruder male was placed in the home cage of each dam with her litter for 60 min between 8:00 a.m. and 15:00 p.m. Intruder males consisted of a group of 20 Sprague Dawley males (240–285 g) that were rotated through the resident females so that each female was always presented with a novel male. Following the introduction of the male intruder, the resident female would investigate the intruder and then attack. Initial confrontations typically consisted of aggressive attacks initiated by the dam (typically boxing or tackling), with intruder males ending up on their backs or retreating away from the nest area attempting to defend themselves. After the initial attack or series of attacks, the female would return to the nest area to care for the pups and/or observe the male. The female would then periodically attack the male during the remainder of the CSS encounter, often depending on whether the male attempted to approach the nest area. As in previous studies of maternal aggression [19,20,48], the use of smaller or similarly sized males ensured consistently submissive behavior from the males throughout the study. Given the previously established effects of the CSS paradigm on maternal behavior [21,22,23], the focus of this study was not to test the effectiveness of the CSS paradigm (stressed *vs.* non-stressed dams), but rather to test the effectiveness of AVP *vs.* saline control in ameliorating the negative behavioral effects of chronic stress.

### 3.3. Behavior Testing

Maternal behavior, which consists of maternal care of the pups and maternal aggression towards a novel intruder, was assessed in all dams during two 30-min tests on days 3, 10 and 17 of lactation. Behavior testing was conducted at randomly selected times between 9:00 a.m. and 12:00 p.m. to avoid habituation effects on behavior. A digital video camera (Panasonic PV-GS180) was used to record maternal behaviors without human interference. Prior to maternal behavior testing, the pups were removed from the home cage for 30 min. The pups were then re-introduced and maternal care was recorded for 30 min (*i.e.*, maternal care test). The latency to retrieve all pups back to the nest, the latency to initiate nursing, as well as the frequencies and durations of pup retrieval, pup grooming, self grooming, nursing, total maternal care (sum of pup grooming and nursing), nesting and general locomotor activity were all scored. Immediately following the maternal care test, a novel intruder male was introduced to the home cage (with both dam and pups present as per the CSS paradigm described above) and maternal aggression was recorded for a subsequent 30 min (thus while dams were exposed to novel male intruders daily for 60 min, only the first 30 min were filmed/observed on behavior testing days). During this maternal aggression test, the latency to initiate nursing, the latency to initiate aggression, as well as the frequencies and durations of pup retrieval, pup grooming, self grooming, nursing, nesting, attacking (frontal and lateral pummeling with forelimbs), biting, kicking with hindlimbs, pinning of intruder to the cage floor, and general locomotor activity were scored. In addition, total maternal care, total aggression (sum of attacking, biting, kicking and pinning), and mean aggressive bout duration (total aggression duration/frequency) were also calculated. All behavior analyses were conducted using ODlog behavioral analysis software (Macropod Inc.) by an observer that was blind to the treatment of each dam. The ODlog software records continuous data in 5 s bins and generates frequency and duration summaries for all behavior measures over the 30-min observation periods. 

Body weights of the dams and pups were recorded on each behavioral testing day to assess if AVP treatment had an overall effect on dam or offspring growth. From these data the percent body weight gain relative to lactation day 3 was calculated for each group on lactation days 10 and 17.

### 3.4. Statistical Analysis

Initial statistical testing of behavioral data was conducted using 2-way repeated measures ANOVAs with lactation day (3, 10 and 17) as the repeated factor (VassarStats). Given that both maternal care and aggression have been previously shown to change over the course of lactation [19,46,48], the primary focus of the current study is to investigate the effects of AVP on maternal behavior; therefore, the effects of lactation day (time) will not be discussed in detail. Following the repeated measures analyses, behavior data collected from saline and AVP treatment groups on lactation days 3, 10 and 17 were analyzed separately with one-way ANOVAs and subsequent one-tailed *t*-tests to assess the effects of AVP on individual lactation days. Due to an absence of dose effects, the individual low and high AVP doses were combined into one overall AVP treatment group. Growth data (mean body weights as well as percent growth relative to lactation day 3) were also compared using one-tailed *t*-tests. All results are presented as the mean ± SEM, and the level of statistical significance was *p* ≤ 0.05.

## 4. Conclusions

In summary, the data support the hypotheses that chronic infusion of AVP promotes maternal care and suppresses maternal aggression during exposure to social stress during early and mid lactation. While the current results from maternal females are not consistent with most male studies, they are consistent with several studies of AVP and maternal behavior that served as the basis for the present study. The most significant implication of the gender differences in the effects of central AVP is in the development of novel treatments for depression and anxiety. AVP antagonism (V1a and V1b receptors) is an active area of preclinical and clinical research on depression and anxiety disorders [25], but recent studies on AVP and maternal behavior consistently indicate that AVP antagonism may not have beneficial effects in females, as endogenous AVP enhances maternal behavior and inhibits aggression. The current conclusions support the earlier work on AVP and maternal behavior and extend the application of these findings to an ethologically relevant model of postpartum depression and anxiety. While it is unclear if increased maternal aggression would be a negative effect of central AVP antagonism, decreases in maternal care would be detrimental, especially in situations where care is already impaired (i.e. postpartum depression). Our conclusions support the focus on AVP as a valid target for the development of novel treatments directed at the adverse behavioral effects of chronic stress associated disorders [77], and emphasize the need for female specific studies on the behavioral roles of AVP.

## References

[1] Patel V.N., DeSouza N., Rodrigues M. (2003). Postnatal depression and infant growth and development in low income countries: A cohort study from Goa, India. Arch. Dis. Child..

[2] Surkan P.J., Kawachi I., Ryan L.M., Berkman L.F., Carvalho Vieira L.M., Peterson K.E. (2008). Maternal depressive symptoms, parenting self-efficacy, and child growth. Am. J. Public Health.

[3] Goodman S.H. (2007). Depression in mothers. Annu. Rev. Clin. Psychol..

[4] Paykel E., Emms E., Fletcher J., Rassaby E. (1980). Life events and social support in puerperal depression. Br. J. Psychiatry.

[5] O’Hara M.W., Rehm L.P., Campbell S.B. (1983). Postpartum depression: A role for social network and life stress variables. J. Nerv. Ment. Dis..

[6] O’Hara M.W. (1986). Social support, life events, and depression during pregnancy and the puerperium. Arch. Gen. Psychiatry.

[7] Seguin L., Potvin L., St-Denis M.L., Loiselle J. (1995). Chronic stressors, social support, and depression during pregnancy. Obstet. Gynecol..

[8] Beck C.T. (1996). A meta-analysis of predictors of postpartum depression. Nurs. Res..

[9] O’Hara M.W., Swain A.M. (1996). Rates and risk of postpartum depression: A meta-analysis. Int. Rev. Psychiatry.

[10] Beck C.T. (2001). Predictors of postpartum depression: An update. Nurs. Res..

[11] Robertson E., Grace S., Wallington T., Stewart D.E. (2004). Antenatal risk factors for postpartum depression: A synthesis of recent literature. Gen. Hosp. Psychiatry.

[12] Klier C.M., Rosenblum K.L., Zeller M., Steinhardt K., Bergemann N., Muzik M. (2008). A multirisk approach to predicting chronicity of postpartum depression symptoms. Depress. Anxiety.

[13] Hillerer K.M., Reber S.O., Neumann I.D., Slattery D.A. (2011). Exposure to chronic pregnancy stress reverses peripartum-associated adaptations: Implications for postpartum anxiety and mood disorders. Endocrinology.

[14] Westdahl C., Milan S., Magriples U., Kershaw T.S., Rising S.S., Ickovics J.R. (2007). Social support and social conflict as predictors of prenatal depression. Obstet. Gynecol..

[15] Maestripieri D., Badiani A., Puglisi-Allegra S. (1991). Prepartal chronic stress increases anxiety and decreases aggression in lactating female mice. Behav. Neurosci..

[16] Léonhardt M., Matthews S.G., Meaney M.J., Walker C.D. (2007). Psychological stressors as a model of maternal adversity: Diurnal modulation of corticosterone responses and changes in maternal behavior. Horm. Behav..

[17] Neumann I., Toschi N., Ohl F., Torner L., Kromer S.A. (2001). Maternal defense as an emotional stressor in female rats: Correlation of neuroendocrine and behavioral parameters and involvement of brain oxytocin. Eur. J. Neurosci..

[18] Douglas A.J., Meddle S.L., Kroemer S., Muesch W., Bosch O.J., Neumann I.D. (2007). Social stress induces hypothalamo-pituitary-adrenal axis responses in lactating rats bred for high trait anxiety. Eur. J. Neurosci..

[19] Nephew B.C., Bridges R.S. (2008). Central actions of arginine vasopressin and a V1a receptor antagonist on maternal aggression, maternal behavior, and grooming in lactating rats. Pharmacol. Biochem. Behav..

[20] Nephew B.C., Bridges R.S., Lovelock D.F., Byrnes E.M. (2009). Enhanced maternal aggression and associated changes in neuropeptide gene expression in reproductively experienced rats. Behav. Neurosci..

[21] Nephew B.C., Bridges R.S. (2011). Effects of chronic social stress during lactation on maternal behavior and growth in rats. Stress.

[22] Murgatroyd C., Carini L., Nephew B.C. (2012). Effects of chronic social stress on maternal behavior, anhedonia, milk intake, pup growth, and gene expression. Eur. J. Psychotraumtol..

[23] Murgatroyd C., Nephew B.C. (2012). Effects of early life stress on maternal behavior and neuroendocrinology. Psychoneuroendocrinology.

[24] Insel T.R., Young L.J. (2000). Neuropeptides and the evolution of social behavior. Curr. Opin. Neurobiol..

[25] Rotzinger S., Lovejoy D.A., Tan L.A. (2010). Behavioral effects of neuropeptides in rodent models of depression and anxiety. Peptides.

[26] Bosch O.J. (2011). Maternal nurturing is dependent on her innate anxiety: The behavioral roles of brain oxytocin and vasopressin. Horm. Behav..

[27] Bosch O.J., Neumann I.D. (2011). Both oxytocin and vasopressin are mediators of maternal are and aggression in rodents: From central release to sites of action. Horm. Behav..

[28] Fleming A.S. (1979). Maternal nest defense in the desert woodrat *Neotoma lepida*. Behav. Neural. Biol..

[29] Wolff J.O. (1985). Maternal aggression as a deterrent to infanticide in Peromyscus leucopus and *P. maniculatus*. Anim. Behav..

[30] Maestripieri D., Alleva E. (1990). Maternal aggression and litter size in the female house mouse. Ethology.

[31] Wolff J.O. (1993). Why are female small mammals territorial?. Oikos.

[32] Vom Saal F.S., Franks P., Boechler M., Palanza P., Parmigiani S. (1995). Nest defense and survival of offspring in highly aggressive wild Canadian female house mice. Physiol. Behav..

[33] Wolff J.O., Peterson J.A. (1998). An offspring-defense hypothesis for territoriality in female mammals. Ethol. Ecol. Evol..

[34] Numan M., Insel T.R. (2003). The Neurobiology of Parental Behavior.

[35] Ferris C.F., Meenan D.M., Axelson J.F., Albers H.E. (1986). A vasopressin antagonist can reverse dominant/subordinate behavior in hamsters. Physiol. Behav..

[36] Ferris C.F., Potegal M. (1988). Vasopressin receptor blockade in the anterior hypothalamus suppresses aggression in hamsters. Physiol. Behav..

[37] Elkabir D.R., Wyatt M.E., Vellucci S.V., Herbert J. (1990). The effects of separate or combined infusions of corticotropin releasing factor and vasopressin either intraventricularly or into the amygdala on aggressive and investigative behavior inthe rat. Regul. Pept..

[38] Compaan J.C., Buijs R.M., Pool C.W., de Ruiter J.H., Koolhaas J.M. (1993). Differential lateral septal vasopressin innervation in aggressive and nonaggressive male mice. Brain Res. Bull..

[39] Delville Y., Mansour K.M., Ferris C.F. (1996). Serotonin blocks vasopressin-facilitated offensive aggression: Interactions within the ventrolateral hypothalamus of golden hamsters. Physiol. Behav..

[40] Delville Y., Mansour K.M., Ferris C.F. (1996). Testosterone facilitates aggression by modulating vasopressin receptors in the hypothalamus. Physiol. Behav..

[41] Ferris C.F., Melloni R.H., Koppel G., Perry K.W., Fuller R.W., Delville Y. (1997). Vasopressin/serotonin interaction in the anterior hypothalamus control aggressive behavior in golden hamsters. Neuroscience.

[42] Stribley J.M., Carter C.S. (1996). Developmental exposure to vasopressin increases aggression in adult prairie voles. Proc. Natl. Acad. Sci. USA.

[43] Caldwell H.K., Lee H.J., Macbeth A.H., Young W.S. (2008). Vasopressin: Behavioral roles of an “original” neuropeptide. Prog. Neurobiol..

[44] Gutzler S.J., Karom M., Albers H.E. A Vasopressin (V1a) Receptor Antagonist Stimulates Aggression in Female Syrian Hamsters, but not during Behavioral Estrus. Presented at the 37th annual meeting of the Society for Neuroscience.

[45] Erskine M.S., Barfield R.J., Goldman B.D. (1978). Intraspecific fighting during late pregnancy and lactation in rats and effects of litter removal. Behav. Biol..

[46] Mayer A.D., Rosenblatt J.S. (1987). Hormonal factors influence the onset of maternal aggression in laboratory rats. Horm. Behav..

[47] Caughey S.D., Klampfl S.M., Bishop V.R., Pfortsch J., Neumann I.D., Bosch O.J., Meddle S.L. (2011). Changes in the intensity of maternal aggression and central oxytocin and vasopressin V1a receptors across the peripartum period in the rat. J. Neuroendocrinol..

[48] Bosch O.J., Neumann I.D. (2010). Vassopressin released through the central amygdala promotes maternal aggression. J. Eur. Neurosci..

[49] Nephew B.C., Byrnes E.M., Bridges R.S. (2010). Vasopressin mediates enhanced offspring protection in multiparous rats. Neuropharmacology.

[50] Caffrey M.K., Nephew B.C., Febo M. (2010). Central vasopressin V1a receptors modulate neural processing in mothers facing intruder threat to pups. Neuropharmacology.

[51] Bosch O.J., Neumann I.D. (2008). Brain vasopressin is an important regulator of maternal behavior independent of dams’ trait anxiety. Proc. Natl. Acad. Sci. USA.

[52] Nephew B.C., Bridges R.S. (2008). Arginine vasopressin V1a receptor antagonist impairs maternal memory in rats. Physiol. Behav..

[53] Pedersen C.A., Caldwell J.D., Walker C., Ayers G., Mason G.A. (1994). Oxytocin activates the postpartum onset of rat maternal behavior in the ventral tegmental and preoptic areas. Behav. Neurosci..

[54] Heinrichs M., Domes G. (2008). Neuropeptides and social behaviour: Effects of oxytocin and vasopressin in humans. Prog. Brain Res..

[55] Scott L.V., Dinan T.G. (1998). Vasopressin and the regulation of hypothalamic-pituitary-adrenal axis function: implications for the pathophysiology of depression. Life Sci..

[56] Nakase S., Kitayama I., Soya H., Hamanaka K., Nomura J. (1998). Increased expression of magnocellular arginine vasopressin mRNA in paraventricular nucleus of stress induced depression-model rats. Life Sci..

[57] Muller M.B., Landgraf R., Keck M.E. (2000). Vasopressin, major depression, and hypothalamic-pituitary-adrenocortical desensitization. Biol. Psychiatry.

[58] Goekoop J.G., de Winter R.P.F., de Rijk R., Zwinderman K.H., Frankhuijzen-Sierevogel A., Wiegant V.M. (2006). Depression with above-normal plasma vasopressin: Validation by relations with family history of depression and mixed anxiety and retardation. Psychiatry Res..

[59] Veenema A.H., Blume A., Niederle D., Buwalda B., Neumann I.D. (2006). Effects of early life stress on adult male aggression and hypothalamic vasopressin and serotonin. Eur. J. Neurosci..

[60] Surget A., Belzung C. (2008). Involvement of vasopressin in affective disorders. Eur. J. Pharmacol..

[61] Scott L.V., Dinan T.G. (2002). Vasopressin as a target for antidepressant development: an assessment of the available evidence. J. Affect. Disord..

[62] Litvin Y., Murakami G., Pfaff D.W. (2011). Effects of chronic social defeat on behavioral and neural correlates of sociality: Vasopressin, oxytocin and the vasopressinergic V1b receptor. Physiol. Behav..

[63] Thompson R.R., George K., Walton J.C., Orr S.P., Benson J. (2006). Sex-specific influences of vasopressin on human social communication. Proc. Natl. Acad. Sci. USA.

[64] Neumann I., Johnston H., Hatzinger M., Liebsch G., Shipston M., Russell J.A., Landgraf R., Douglas A.J. (1998). Attenuated neuroendocrine responses to emotional and physical stressors in pregnant rats involve adenohypophysial changes. J. Physiol..

[65] Douglas A.J., Brunton P.J., Bosch O.J., Russell J.A., Neumann I.D. (2003). Neuroendocrine responses to stress in mice: Hyporesponsiveness in pregnancy and partuition. Endocrinology.

[66] Landgraf R., Wigger A., Holsboer F., Neumann I.D. (1999). Hyperreactive hypothalamo-pituitary-adrenocortical (HPA) axis in rats bred for high anxiety-related behavior (rapid communication). J. Neuroendocrinol..

[67] Keck M.E., Wigger A., Welt T., Müller M.B., Gesing A., Reul J.M., Holsboer F., Landgraf R., Neumann I.D. (2002). Vasopressin mediates the response of the combined dexamethasone/CRH test in hyper-anxious rats: implications for pathogenesis of affective disorders. Neuropsychoparmacology.

[68] Keck M.E., Welt T., Müller M.B., Uhr M., Ohl F., Wigger A., Toschi N., Holsboer F., Landgraf R. (2003). Reduction of hypothalamic vasopressinergic hyperdrive contributes to clinically relevant behavioral and neuroendocrine effects of chronic paroxetine treatment in a psychopathological rat model. Neuropsychoparmacology.

[69] Murgatroyd C., Wigger A., Frank E., Singewald N., Bunck M., Holsboer F., Landgraf R., Spengler D. (2004). Impaired repression at a vasopressin promoter polymorphism underlies overexpression of vasopressin in a rat model of trait anxiety. J. Neurosci..

[70] Wigger A., Sanchez M.M., Mathys K.C., Ebner K., Frank E., Liu D. (2004). Alterations in central neuropeptide expression, release, and receptor binding in rats bred for high anxiety: Critical role of vasopressin. Neuropsychopharmacology.

[71] Neumann I.D., Krömer S.A., Bosch O.J. (2005). Effects of psycho-social stress during pregnancy on neuroendocrine and behavioral parameters in lactation depend on the genetically determined stress vulnerability. Psychoneuroendocrinology.

[72] Crowley R.S., Insel T.R., O’Keefe J.A., Amico J.A. (1993). Cytoplasmic oxytocin and vasopressin gene transcripts decline postpartum in the hypothalamus of the lactating rat. Endocrinology.

[73] Champagne F.A., Francis D.D., Mar A., Meaney M.J. (2003). Variations in maternal care in the rat as a mediating influence for the effects of environment on development. Physiol. Behav..

[74] Adewuya A.O., Ola B.O., Aloba O.O., Mapayi B.M., Okeniyi J.A.O. (2008). Impact of postnatal depression on infants’ growth in Nigeria. J. Affect. Disord..

[75] Lau C. (2001). Effects of stress on lactation. Pediatr. Clin. North Am..

[76] De Goeij D.C., Dijkstra H., Tilders F.J. (1992). Chronic psychosocial stress enhances vasopressin, but not corticotropic-releasing factor, in the external zone of the median eminence of male rats: Relationship to subordinate status. Endocrinology.

[77] Meyer-Lindenberg A., Domes G., Kirsch P., Heinrichs M. (2011). Oxytocin and vasopressin in the human brain: Social neuropeptides for translational medicine. Nat. Rev. Neurosci..

